# Towards a regional and national cardiothoracic biobank in India: translating a Dutch model to address a poorly characterised disease burden

**DOI:** 10.1016/j.lansea.2026.100860

**Published:** 2026-09-10

**Authors:** Nimrat Grewal, Mohammed Idhrees, Bashi Velayudhan, Mohamad Bashir

**Affiliations:** a Department of Cardiothoracic Surgery, Amsterdam University Medical Center location AMC, Meibergdreef 9, 1105 AZ, Amsterdam, the Netherlands; b Aortic Institute at Yale New Haven Hospital, Yale University School of Medicine, 333 Cedar St, New Haven, CT, 06510, USA; c Institute of Cardiac and Aortic Disorders (ICAD), SRM Institutes for Medical Science (SIMS Hospital), Chennai, India; d Vascular & Endovascular Surgery, Velindre University NHS Trust, Health Education & Improvement Wales (HEIW), Cardiff, UK

**Keywords:** Biobank, Cardiothoracic surgery, Aortic dissection, Rheumatic heart disease, Precision medicine, Genomics, Ethical governance, India, Health equity

## Abstract

Cardiothoracic disease in India presents earlier and against a different biological background than in the high-income populations from which most evidence and guidelines derive. Coronary disease occurs about a decade earlier in South Asians; rheumatic heart disease remains endemic, with India carrying a quarter to a half of the global burden; and acute type A aortic dissection presents in the mid-to-late forties, with a higher reported proportion of connective-tissue disease than Western registries. The biological substrate of these conditions in Indian patients is largely uncharacterised, and South Asian populations are underrepresented in the datasets driving precision cardiovascular medicine. We argue that a prospectively designed, ethically governed cardiothoracic biobank, collecting surgical tissue, blood and longitudinal clinical data at operation, is foundational national research infrastructure, and we describe what it can deliver, a transferable Dutch model (the IHLCN biobank), an ethical governance and data-transfer framework, and a phased regional-to-national path.

Cardiovascular disease is now the leading cause of death in India, and the burden falls on a younger, working-age population than almost anywhere else in the world,^1^ with a first myocardial infarction at a mean age of around 53 years.^2^ These are associations, not fixed biological traits: ethnicity is a sociocultural construct, and the socioeconomic and structural factors for which it may act as a surrogate remain incompletely accounted for.

Still, the pattern is not simply more disease but a different one, emerging five to ten years earlier, often against insulin resistance, central adiposity and an atherogenic lipid profile incompletely explained by conventional risk factors.^3^

Yet the risk scores, diagnostic thresholds and even device dimensions used in Indian operating theatres are calibrated to European and North American populations, not to the younger patient on the table,^4^, ^5^, ^6^ and almost none of the material that could explain their disease is collected.

Exploring this gap is both a scientific opportunity and an ethical obligation. The instrument capable of closing it is well established in high-income systems but largely absent in India for cardiothoracic disease: a disease-focused biobank.

The broad case for integrated, population-scale biobanking in South Asia has recently been made in this journal.^7^ Our proposal is complementary and disease-specific: alongside the blood, imaging and longitudinal outcome data any biobank collects, a cardiothoracic surgical biobank adds the diseased tissue itself, aorta, valve and myocardium removed at operation, linked to long-term outcome, which no existing South Asian resource captures (Table 1). It could serve as a disease-specific node within the regional consortium those authors envisage, supplying the mechanistic layer a population-scale infrastructure lacks.

**Table 1:** The Indian biobanking landscape and the cardiothoracic gap.

Existing and planned national resources are compared with the proposed cardiothoracic biobank and its Dutch template. No existing Indian resource collects diseased cardiothoracic tissue or links it to surgical outcome; the two shaded rows are the disease-specific, tissue-based resources.

CSIR-IGIB, CSIR-Institute of Genomics and Integrative Biology; DBT, Department of Biotechnology; IHLCN, Instituut Hart Long Chirurgie Nederland.

Three surgical conditions show how far the Indian pattern departs from the populations that dominate the literature. South Asians carry one of the world's highest burdens of premature coronary artery disease, presenting about a decade earlier than Western cohorts, yet its proteomic, metabolic and genetic drivers have scarcely been catalogued in tissue or blood taken at revascularisation; coronary dimensions are also smaller, so grafts and sizing thresholds derived elsewhere may not transfer directly.^8^, ^9^, ^10^ Rheumatic heart disease, largely eliminated in high-income countries, remains endemic, India carries an estimated 25–50% of the global burden, concentrated in children, adolescents and young women, and drives much of the country's valve surgery, yet attracts little international interest; valve and myocardial tissue removed at operation, paired with longitudinal data, is an irreplaceable and currently discarded resource.^3^^,^^11^ Acute type A aortic dissection presents at a mean age of about 48 years, far younger than international registries, with Marfan syndrome in roughly 14% of patients, over three times the IRAD proportion, signalling a heritable, connective-tissue–driven aortopathy that demands aortic wall tissue, paired skin biopsies and linked genetic and imaging data to understand.^12^, ^13^, ^14^, ^15^, ^16^

Precision cardiovascular medicine sharpens the case. Biobanks such as UK Biobank show what becomes possible when tissue, blood, imaging, genomics and outcomes are linked at scale, validated polygenic risk scores, deep-learning aortic phenotyping and plasma proteomics nominating hundreds of drug targets.^17^^,^^18^ Yet over 80% of the underpinning genomic data derive from individuals of European ancestry.^19^ GenomeIndia has begun to close this gap (nearly 10,000 genomes across 83 populations),^20^ and in July 2025 the Phenome India National Biobank, modelled on UK Biobank, was inaugurated at CSIR-IGIB,^21^ alongside mainly oncological institutional biobanks in several cities.^22^ But these are population-genomics and general-cohort resources, not disease-linked surgical biobanks: they describe the background against which cardiothoracic disease develops, yet cannot link a genotype to the diseased aorta, valve or myocardium removed at surgery, or to surgical outcome. The two are complementary, and India, at present, has only one of them (Table 1).

Such a biobank maps onto each gap above: surgical tissue with paired skin biopsies and stem-cell models to dissect premature coronary disease, rheumatic valve damage and early-onset aortopathy; blood, plasma and tissue linked to outcomes to yield biomarkers of progression; and multi-omic profiling for India-calibrated risk models,^17^ helping correct the underrepresentation of South Asians in the data defining cardiovascular care.^19^

We do not propose to start from a blank page. One of us (NG) has established and obtained ethical approval for a comprehensive cardiothoracic biobank in the Netherlands—the Biobank Cardiothoracic Surgery/Instituut Hart Long Chirurgie Nederland (IHLCN biobank) at Amsterdam UMC, which can serve directly as a design template (Table 1).^23^^,^^24^ Its design is deliberately pragmatic and transferable:

Its design is deliberately pragmatic and transferable. Collection is embedded in routine care, no extra visit or separate venipuncture, with blood drawn during scheduled pre-operative sampling and tissue taken from material removed at the planned operation. Sampling is broad but high-value: blood across plasma, DNA, PBMC, serum and RNA fractions; a skin biopsy for cell-line work; and residual valve, aortic wall, myocardium, pericardium and vessel tissue, with optional intra-operative sublingual microcirculation imaging.^25^ Standardised peri-operative variables and patient-reported outcomes run to 5–10 years in a coded database. The protocol targets around 1200 participants per centre per year, readily matched by high-throughput Indian units, though accrual should track what can realistically be stored, curated and used. Crucially, the protocol, patient information, consent and withdrawal forms, data dictionary and SOPs already exist in mature, ethics-approved form, so translation to India is largely legal and cultural adaptation and regulatory harmonisation, not de novo design.

We propose a phased architecture (Fig. 1). A regional pilot at one or two high-volume tertiary cardiac centres would establish the consent workflow, sample-handling SOPs, cold-chain and storage, and the coded clinical database, and prove feasibility.

**Fig. 1:**
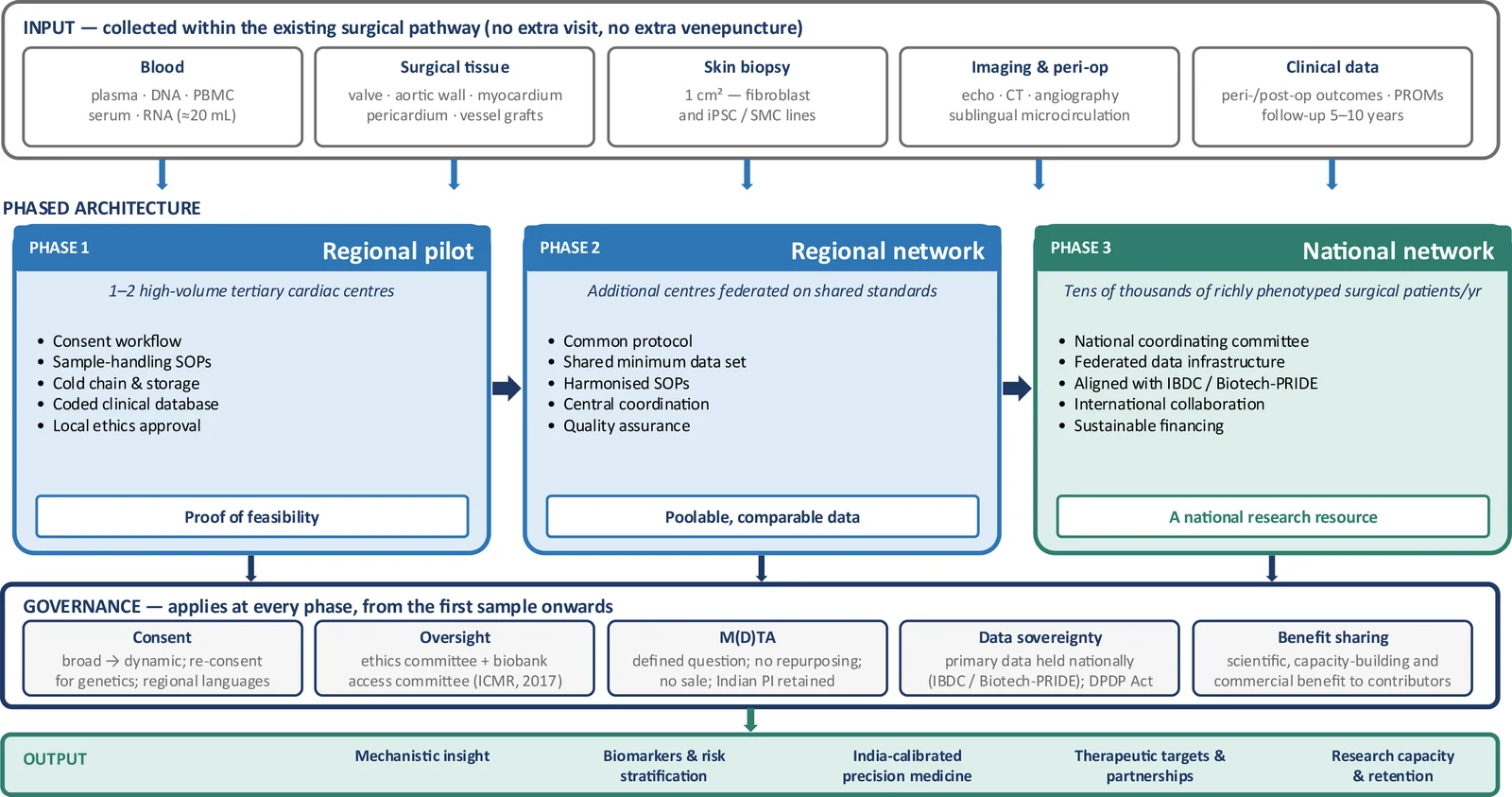
Architecture of a proposed regional-to-national cardiothoracic biobank in India. Material is collected within the existing surgical pathway, requiring no additional hospital visit or venipuncture. The biobank scales in three phases, from a regional pilot to a national network. Governance applies from the first sample onwards rather than being added later. iPSC, induced pluripotent stem cell; SMC, smooth muscle cell; PROM, patient-reported outcome measure; M(D)TA, Material (and Data) Transfer Agreement; IBDC, Indian Biological Data Centre; ICMR, Indian Council of Medical Research; DPDP Act, Digital Personal Data Protection Act 2023.

A national network would then federate additional centres around these shared standards, governed by a coordinating committee and aligned with India's emerging framework, the ICMR National Ethical Guidelines^26^ and the Biotech-PRIDE guidelines, implemented through the Indian Biological Data Centre under the Department of Biotechnology.^27^

A biobank earns trust through its governance, not only its science, especially in low- and middle-income settings, where weak governance and the absence of clear transfer agreements have historically let biological material and the patients’ own interests slip beyond local control.^28^

Several core requirements are well described internationally and need not be stated^28^, ^29^, ^30^, ^31^: broad consent that can evolve toward dynamic, re-contactable consent, with specific re-consent for germline analysis; independent ethics and access committees overseeing every release; and no sample or dataset leaving the custodian institution except under a Material (and Data) Transfer Agreement that fixes the research question, retains an Indian principal investigator, prohibits repurposing, re-identification and sale, and specifies benefit-sharing. Two issues are specific to India.

The first is community involvement: India's cultural, religious and regional diversity means people legitimately hold differing views on biobanking, strengthening the case for dedicated patient and public involvement, regional advisory groups, for example from the rheumatic heart disease and young surgical populations, should shape consent and build trust, with materials translated into the major regional languages. The second is data sovereignty: genomic and clinical data should be governed under Biotech-PRIDE and the Indian Biological Data Centre, keeping primary data within national infrastructure,^27^ with benefits flowing back to contributing institutions and communities and Indian investigators leading analysis of Indian patients' material.^28^ India still lacks a dedicated genomic data-protection statute for biobanks and datasets such as IndiGen and GenomeIndia; this incompleteness is itself the argument for carrying participant protection in the biobank's own architecture, consent model, access committee, M(D)TA and national data custody, so a resource built to anticipate a future statute need not be retrofitted.

Commercial partnership deserves particular care, as public–private partnership is actively promoted in Indian health policy. Well governed, industry involvement is an opportunity, not a threat: it should proceed only for a defined research question, under an M(D)TA with an institutional investigator, explicit consent and benefit-sharing, never by outright sale of samples, the model already operationalised in the IHLCN biobank.^24^

Two practical obstacles deserve candour. The first is sustainable funding: biobanks are long-term commitments whose value compounds over years, and they require a financing model, blending public research funding, philanthropy, hospital investment, and carefully governed industry partnership, that can outlast any single grant; financial sustainability is the challenge most consistently reported by biobanks in comparable settings.^28^ The second is operational: sample logistics, cold-chain integrity, storage, and data security demand professional infrastructure and trained staff. Data should routinely be disaggregated and reported by sex and gender.

India treats one of the largest, youngest, and most biologically distinctive cardiothoracic surgical populations in the world, and currently discards almost all of the biological material that could explain their disease and improve their care. The scientific tools to exploit such material have never been more powerful, yet South Asians remain among the most underrepresented populations in the data driving cardiovascular precision medicine. A regional, and ultimately national, cardiothoracic biobank, built on a proven, ethically approved model and governed in the interests of Indian patients, is a foundational piece of research infrastructure whose absence is increasingly difficult to justify. The main barrier is not feasibility but sustained funding and coordination.

## Contributors

NG conceived the Viewpoint and wrote the first draft. MI, BV, and MB contributed to the concept, provided India-specific content and critically revised the manuscript. All authors reviewed and approved the final version. NG was responsible for the decision to submit. As a Viewpoint, the article reports no primary data requiring verification.

## Declaration of interests

We declare no competing interests.
